# Clinical variables of preoperative risk in thoracic surgery

**DOI:** 10.1590/S1516-31802003000300004

**Published:** 2003-05-01

**Authors:** Ivete Alonso Bredda Saad, Eduardo Mello De Capitani, Ivan Felizardo Contrera Toro, Lair Zambon

**Keywords:** Thoracic surgical procedures, Postoperative period, Postoperative complications, Pulmonary surgical procedures., Procedimentos cirúrgicos torácicos, Período pós-operatório, Complicações pós-operatória, Procedimentos cirúrgicos pulmonares

## Abstract

**CONTEXT:**

Pulmonary complications are the most common forms of postoperative morbidity in thoracic surgery, especially atelectasis and pneumonia. The first step in avoiding these complications during the postoperative period is to detect the patients that may develop them.

**OBJECTIVE:**

To identify risk variables leading to early postoperative pulmonary complications in thoracic surgery.

**DESIGN:**

Prospective study.

**SETTING:**

Hospital das Clínicas, Faculdade de Ciências Médicas, Universidade Estadual de Campinas.

**PATIENTS:**

145 patients submitted to elective surgery were classified as low, moderate and high risk for postoperative pulmonary complications using a risk assessment scale.

**PROCEDURES:**

The patients were followed up for 72 hours after the operation. Postoperative pulmonary complications were defined as atelectasis, pneumonia, tracheobronchitis, wheezing, prolonged intubation and/or prolonged mechanical ventilation.

**MAIN MEASUREMENTS:**

Univariate analysis was applied in order to study these independent variables: age, nutritional status, body mass index, respiratory disease, smoking habit, spirometry and surgery duration. Multivariate logistic regression analysis was performed in order to evaluate the relationship between independent and dependent variables.

**RESULTS:**

The incidence of postoperative complications was 18.6%. Multivariate logistic regression analysis showed that the variables increasing the chances of postoperative pulmonary complications were wheezing (odds ratio, OR = 6.2), body mass index (OR = 1.15), smoking (OR = 1.04) and surgery duration (OR = 1.007).

**CONCLUSION:**

Wheezing, body mass index, smoking and surgery duration increase the chances of postoperative pulmonary complications in thoracic surgery.

## RESUMO

**CONTEXTO:**

As complicações pulmonares pósoperatórias, principalmente a pneumonia e atelectasia, têm sido freqüentes causas de morbidade nas cirurgias torácicas. Há interesse em se identificar fatores no processo de avaliação pré-operatória que possam, isoladamente ou associados, contribuir para o aparecimento de tais complicações.

**OBJETIVO:**

Identificar as variáveis de risco para complicação pulmonar pós-operatórias na cirurgia torácica.

**TIPO DE ESTUDO:**

Estudo prospectivo.

**LOCAL:**

Hospital das Clínicas da Universidade Estadual de Campinas

**PARTICIPANTES:**

145 pacientes adultos, ambos os sexos, internados para cirurgia torácica eletiva e classificados em baixo, moderado e alto risco para complicação pulmonar pósoperatória utilizando-se uma escala de avaliação de risco.

**PROCEDIMENTO:**

Os pacientes foram acompanhados até as 72 horas do pós-operatório e avaliados quanto ao aparecimento de complicações pulmonares pós-operatória, definidas com o aparecimento de atelectasia, pneumonia, traqueobronquite, chiado, intubação ou ventilação mecânica prolongada.

**VARIÁVEIS ESTUDADAS:**

A análise univariada foi aplicada no estudo das variáveis independentes idade, estado nutricional, doença respiratória, hábitos tabágicos, espirometria e tempo cirúrgico. A análise dos resultados através da regressão logística multivariada mostrou relação entre as variáveis dependentes e independentes.

**RESULTADOS:**

As complicações pulmonares pós-operatórias incidiram sobre 18.6% da amostra. Através da regressão logística multivariada, verificou-se que as variáveis que aumentaram as chances de complicações pulmonar pós-operatórias foram chiado (Odds ratio, OR = 6.2), estado nutricional (OR = 1.15), tabagismo (OR = 1.04) e duração da cirurgia (OR = 1.007).

**CONCLUSÃO:**

Chiado, estado nutricional, tabagismo e duração da cirurgia aumentaram a ocorrência de complicações pulmonares pós-operatórias em cirurgia torácica.

## INTRODUCTION

The most common forms of postoperative morbidity in thoracic surgery are pulmonary complications, especially atelectasis and pneumonia.^1-7^ The first step towards avoiding these complications, which should be taken during the postoperative period, is to detect the patients at risk of developing them.

Most of the clinical factors that are responsible for these complications result from pulmonary alterations and can be divided into four categories: a) mechanical pulmonary alterations; b) alterations in the respiratory pattern; c) alterations in gaseous exchange; d) alterations in the pulmonary defense mechanism.^1,7-10^ These dysfunctions are not limited to patients with lung diseases and can occur in any individual during the postoperative period .^11,12^

The risk factors that increase the chances of postoperative pulmonary complications are respiratory diseases, smoking habit, nutritional status, age and surgery duration. As respiratory diseases are important risk factors for pulmonary complications, the routine procedure in elective surgery is to wait for the process to stabilize.^7,13-15^ Even in those patients who do not suffer from any pulmonary disease, smoking is a risk factor for postoperative pulmonary complications. The patient should stop smoking at least 8 weeks before the surgery, in order to reduce complications.^1,16-18^

Obese patients are susceptible to ineffective cough, atelectasis at the base of the lungs and progressive hypoxia that facilitates the onset of infections. Malnutrition, on the other hand, is also an important risk factor because protein-calorie deficiency induces alterations in pulmonary defenses.^19-21^

The main spirometric alterations resulting from lung surgery are related to vital capacity and functional residual capacity. Following major operations, the vital capacity diminishes by 50% to 60% and functional residual capacity by 30% over the first 16 to 24 hours after the operation, regaining their normal values by the fifth postoperative day.^1,20^ Spirometry may indicate the presence and severity of the pulmonary disease, but in isolation it does not offer enough information to predict the risk of pulmonary complications.^7,17,22^ In the case of asymptomatic respiratory patients, its performance as an indicator is debatable.^23^

One of the risk factors to be ascertained during the preoperative assessment is the duration of the surgery. According to some authors, surgery lasting for more than 210 minutes is a risk factor for postoperative pulmonary complications.^7,20,24^

The incidence of postoperative pulmonary complications in thoracic surgeries ranges from 12% to 50%, depending on the criteria evaluated.^6,17,24-27^ This variation comes mainly from the differences in defining diagnostic criteria for postoperative complications in the face of physiopathological disorders particular to the condition and type of surgery.^11,28,29^ The most common complications that occur during the postoperative period involve the development of a restrictive pattern of pulmonary dysfunction related to atelectasis and reduced diaphragmatic movement.^30,31^ The most frequently cited complications are tracheobronchial infection, pneumonia, atelectasis, bronchospasm, prolonged mechanical ventilation and acute respiratory insufficiency.^17,22,26,30^

The objective of this study was to detect the preoperative variables that give rise to a risk of postoperative pulmonary complications in patients who have undergone thoracic surgery.

## METHOD

This prospective study of cohorts was conducted at the Hospital das Clínicas, Universidade Estadual de Campinas (Unicamp), and was approved by the Committee for Ethics in Research of Unicamp. It involved 145 adult patients of both sexes, ranging from 20 to 78 years of age, who underwent elective thoracic surgery between October 1995 and June 1999, with postoperative follow-up lasting 72 hours. For the whole group, preventive measures were adopted regarding the cessation of smoking, use of prophylactic antibiotic therapy when necessary and physical therapy, with the aim of minimizing the risks of complications.

The pre and postoperative assessment questionnaire included: 1) pulmonary history; 2) preoperative physical examination;^32-34^ 3) complementary tests – laboratory tests, lung imaging and pulmonary function examination using forced vital capacity percentile values, forced expiratory volume at the first second and the percentile relationship between them; 4) assessment of the risk of postoperative pulmonary complications – the PORT (Perioperative Respiratory Therapy) risk scale devised by Torrington & Henderson (1988)^8^ was used (Table 1). The PORT scale classifies risk into low (1), moderate (2) and high (3). This latter is a program that evaluates the individual's postoperative risks and care to be taken. The protocol evaluates the following: site of surgery, age, nutritional status, pulmonary history and spirometry. Patients are given scores and classified as being at low, moderate or high risk of postoperative pulmonary complications. Information on surgery duration is also evaluated.

**Table 1:** Torrington & Henderson Scale (1988) Score

CLINICAL FACTORS		SCORE
1. Site of surgery: Thorax		2
2. Age over 65 years		1
3. Nutritional condition – dystrophic		1
4. Pulmonary history		
Smoking		1
Pulmonary disease		1
Cough + expectoration/bronchospasm/hemoptysis		1
5. Spirometry		
FVC < 50% of estimate		1
or FEV_1_/FVC	65-75%	1
	50-65%	2
	< 50%	3
**Low risk – 0 to 3; Moderate risk – 4 to 6; High risk – 7 to 12.**		

*FVC = forced vital capacity; FEV_1_ = forced expiratory volume at the first second.*

Postoperative evolution observations were noted down up to the 3^rd^ postoperative day to check for the following pulmonary complications: acute respiratory infection; atelectasis with clinical repercussion; acute respiratory insufficiency; orotracheal intubation and/or prolonged mechanical ventilation; and bronchospasm. The artificial respirator used in the thoracic operations was the Takaoka Fuji, model 675.

All patients received physiotherapy, such as measures to prevent and treat respiratory dysfunction during the hospitalization.

The Epi-Info 6.04 program was used to store the data for statistical analysis. The chi-squared test and the Fisher Exact test were used to conduct a comparative analysis. The backward multivariate regression analysis that is part of the SAS/STAT statistical software package was then used. This procedure allowed adjustment of the independent variables to be made in relation to the dependent variables, by means of the best combination for explaining the phenomenon observed (complicated/not complicated). The results demonstrated what the participation of each independent variable in this study was, via the odds ratio and 95% confidence interval. In all the tests, the statistical significance level adopted was 5% (p < 0.05).^35,36^

## RESULTS

The study involved 145 patients, of whom 93 were males (64.1%), and 106 of these patients underwent pulmonary parenchyma re-section. The most frequent surgical procedures were lobectomy, for 70 patients (48.2%), followed by pneumonectomy, for 30 patients (20.7%). The most common disease was pulmonary carcinoma, with 89 patients (61.3%).

With regard to age and pulmonary complications, 17 patients (62.9%) were under the age of 65 years, of whom 13 were males (76.5%) and 4 were females (23.5%).

Of the 145 patients who underwent surgery, 27 patients (18.6%) presented pulmonary complications during the 72-hour postoperative period, and 23 (15.9%) of them had undergone pulmonary parenchyma resection. The single most common form of complication was tracheobronchitis, in 29.6% (8/27), followed by bronchospasm in 14.8% (4/27).

The average body mass index (BMI) of the patients with postoperative pulmonary complications was 22.2 kg/m^2^ (standard deviation, SD = 43.5). Seventeen patients were eutrophic (62.9%) and ten patients (37.1%) were dystrophic, of whom nine (33.3%) were undernourished and one (3.7%) obese.

The preoperative complaints of the 145 patients were: coughing in 81 patients (55.8%); dyspnea in 55 patients (37.9%); and wheezing in 22 patients (15.1%). In the cases of the 27 patients with postoperative pulmonary complications, the complaints were: coughing in 20 patients (74%), dyspnea in 13 patients (48.1%); and wheezing in 7 patients (25.9%). The only symptom that demonstrated statistical significance was coughing (chi-squared test; p = 0.02).

Of the 27 patients with postoperative pulmonary complications, 26 patients (96.2%) had a history of smoking; 14 patients (51.8%) were current smokers.

An analysis of the group that presented postoperative pulmonary complications showed that in the cases of 24 patients (88.8%) the surgery took more than 210 minutes and in the cases of 3 patients the surgery took less than 210 minutes. This difference was statistically significant.

The PORT risk scale was applied to the 27 cases that developed complications and it was found that only one patient (3.7%) was low risk; 17 (62.9%) were moderate risk patients; 9 patients (33.3%) were high risk (chi-squared test; p = 0.001.)

The multivariate logistic regression analysis of the independent risk variables for developing postoperative pulmonary complications took into consideration the clinical variables, the length of surgery (Table 2), and also the PORT scale (Table 3), and the following results were obtained:

**Table 2:** Logistic regression analysis between independent and dependent variables in thoracic surgery

Variable	Estimated parameter	P	Odds ratio	Confidence interval
Intercept	- 1.2548	0.4899	-	-
Body mass index	- 0.1437	0.0328	0.87	0.76 - 0.99
Smoking	0.0397	0.0029	1.04	1.01 - 1.07
Wheezing	1.8352	0.0209	6.27	1.32 - 29.7
Duration	0.00686	0.0288	1.01	1.00 - 1.01

*Individuals classified according to the PORT (Perioperative Respiratory Therapy) scale as being at moderate risk had four times the chance of having postoperative pulmonary complications, in comparison with the low-risk classification. The high-risk classification had 22 times the chance of those at moderate risk.*

**Table 3:** Logistic regression analysis considering the independent variable as the risk

Variable	Estimated parameter	P	Odds ratio	Confidence interval
Intercept	- 4.4876	0.002	-	-
Moderate risk	1.4310	0.19	4.18	0.49 – 35.58
High risk	2.7308	0.02	22.5	1.51 – 155.62

*The model with only the risk variable could not be used for prediction, as the adjustable statistics were not significant. The removal of pulmonary parenchyma and spirometry did not present statistical significance.*

For patients with a history of bronchospasm, the risk of complications increased 6.2-fold; for every one-unit reduction in body mass index (in kilograms), the risk increased 1.15-fold (1/0.886); and for every year of smoking it increased 1.04-fold.

For every one-unit increase in surgery duration (in minutes), the risk of complications increased 1.007-fold.

## DISCUSSION

The factors that are most closely related to the development of postoperative pulmonary complications are probably the surgical incision site, history of pulmonary disease, smoking, obesity, malnutrition, age and surgery duration.^1,3,11,18,24,31,37^

The incidence of immediate postoperative pulmonary complications in the population studied was 18.6%. The most common complication was tracheobronchitis (44.4%). This can be explained by the fact that this sample consisted of a high percentage of patients with lung diseases and a history of smoking. An elevated incidence of pulmonary infection has also been verified by other authors.^9-17^

The patients in the under-65 age group presented a higher incidence of complications. This observation can probably be explained because this group had a greater number of individuals with pulmonary co-morbidity and associated clinical factors. According to data from Torrington & Henderson (1988),^8^ patients aged over 65 years presented a risk factor for postoperative pulmonary morbidity. However, according to the studies of Smetana (1999)^17^ and Williams-Russo et al. (1992),^22^ age as a single factor was not a risk predictor, although in association with other factors it was. These authors considered that postoperative pulmonary complications were more related to the coexistence of diseases, and their findings were similar to ours.

Although obesity has been cited as a risk factor, the group of undernourished patients in this study presented a higher number of complications in comparison with the group of eutrophic and obese individuals. This fact was most probably related to the greater quantity of neoplasia and the consequences of malnutrition.

Respiratory symptoms like dyspnea, coughing and wheezing were important factors in the complications in this sample. The analyses of the data obtained on respiratory diseases and respiratory symptoms were found to overlap. Other authors have demonstrated that the coexistence of respiratory disease and respiratory symptomatology increased the chances of developing postoperative pulmonary complications two or threefold.^15,37^

The incidence of postoperative pulmonary complications in patients with a history of smoking in comparison with those who had never smoked (26/1) was significant and concurred with the data in the literature.^4,24^ Al-though the surgery in our sample was elective, 64 patients (44.1%) did not abandon the smoking habit until 8 weeks before the surgery. Only nine patients really stopped smoking.

Spirometry did not help identify patients with a higher chance of postoperative pulmonary complications. According to Williams-Russo et al.,^22^ spirometric tests did not identify postoperative morbidity in non-thoracic surgery.

The duration of the surgery was greater than 210 minutes for more than 50% of this sample. Among the group with postoperative pulmonary complications, 25 patients had the duration of their surgery prolonged. Surgery duration is an important risk factor for postoperative pulmonary complications. According to Latimer et al.,^15^ Tisi^7^ and Bluman et al .,^24^ a duration of more than 210 minutes is considered a risk factor for postoperative pulmonary complications. On the other hand, Chumillas et al.^37^ analyzed 81 patients who underwent upper abdominal surgery and found a complication rate of 13.6%, and in most of these cases the surgery took more than 120 minutes.

## CONCLUSION

Logistic regression analysis showed that in thoracic surgery, the variables responsible for postoperative pulmonary complications were a history of bronchospasm (Odds ratio, OR = 6.2); reduction in body mass index (in one-kilogram units) (OR = 1.15); every year of smoking (OR = 1.04); increase in the length of surgery (in one-minute units) (OR) =1.007. Although the PORT scale does not predict complications, it helps in the clinical assessment of surgical patients at higher risk of postoperative complications.
